# Every antibiotic, every day: Maximizing the impact of prospective audit and feedback on total antibiotic use

**DOI:** 10.1371/journal.pone.0178434

**Published:** 2017-05-31

**Authors:** Tonya J. Campbell, Melissa Decloe, Suzanne Gill, Grace Ho, Janine McCready, Jeff Powis

**Affiliations:** 1 Division of Infectious Diseases, Michael Garron Hospital, Toronto, Ontario, Canada; 2 Department of Medicine, University of Toronto, Toronto, Ontario, Canada; Azienda Ospedaliera Universitaria di Perugia, ITALY

## Abstract

**Background:**

The success of antimicrobial stewardship is dependent on how often it is completed and which antimicrobials are targeted. We evaluated the impact of an antimicrobial stewardship program (ASP) in three non-ICU settings where all systemic antibiotics, regardless of spectrum, were targeted on the first weekday after initiation.

**Methods:**

Prospective audit and feedback (PAAF) was initiated on the surgical, respiratory, and medical wards of a community hospital on July 1, 2010, October 1, 2010, and April 1, 2012, respectively. We evaluated rates of total antibiotic use, measured in days on therapy (DOTs), among all patients admitted to the wards before and after PAAF initiation using an interrupted time series analysis. Changes in antibiotic costs, rates of *C*. *difficile* infection (CDI), mortality, readmission, and length of stay were evaluated using univariate analyses.

**Results:**

Time series modelling demonstrated that total antibiotic use decreased (± standard error) by 100 ± 51 DOTs/1,000 patient-days on the surgical wards (p = 0.049), 100 ± 46 DOTs/1,000 patient-days on the respiratory ward (p = 0.029), and 91 ± 33 DOTs/1,000 patient-days on the medical wards (p = 0.006) immediately following PAAF initiation. Reductions in antibiotic use were sustained up to 50 months after intervention initiation, and were accompanied by decreases in antibiotic costs. There were no significant changes to patient outcomes on the surgical and respiratory wards following intervention initiation. On the medical wards, however, readmission increased from 4.6 to 5.6 per 1,000 patient-days (p = 0.043), while mortality decreased from 7.4 to 5.0 per 1,000 patient-days (p = 0.001). CDI rates showed a non-significant declining trend after PAAF initiation.

**Conclusions:**

ASPs can lead to cost-effective, sustained reductions in total antibiotic use when interventions are conducted early in the course of therapy and target all antibiotics. Shifting to such a model may help strengthen the effectiveness of ASPs in non-ICU settings.

## Introduction

Antibiotic overuse has led to increasing rates of antibiotic resistant bacterial infections and antimicrobial-related adverse events, resulting in increased patient morbidity and mortality [1]. In response to this pressing public health issue, there has been an increased focus on the judicious use of antibiotics. One strategy to reduce antimicrobial consumption is through hospital-based Antimicrobial Stewardship Programs (ASPs). ASPs aim to optimize the use of antimicrobials through a set of coordinated interventions in order to attenuate the harms of antimicrobial overuse [2].

A recent systemic review and meta-analysis demonstrated that ASPs lead to reductions in total antibiotic use within hospitals as well as antibiotic acquisition costs. Although the strategies utilized were varied, most successful ASP programs incorporated prospective audit and feedback (PAAF) as a critical component [3]. The magnitude of the reduction in total antibiotic use was dependent on the location of the ASP program, with larger reductions noted in ASPs within Intensive Care Units (ICU) [3]. Of the studies evaluating total antibiotic use outside of the ICU, there was substantial variation in their ASP interventions and study designs [4–8]. These studies rarely used control groups, performed PAAF infrequently, had short follow-up, delayed PAAF until microbiological information was available, or targeted only specific antibiotics. A study by Palmay et al. [8] involved the most rigorous study methodology and utilized a 3-day delayed PAAF focusing only on specific targeted antibiotics. The intervention was associated with a large reduction in targeted antimicrobial utilization among patients who met stewardship review criteria. There was no significant change, however, in targeted antibiotic use among all admitted patients, nor was there a change in total antibiotic use.

Although existing literature highlights the importance of PAAF, its impact is likely dependent on how frequently it is completed, how quickly after antibiotic prescription it is initiated, and which antibiotics are targeted [3,8]. Every systemic antibiotic on everyday has the potential to lead to antimicrobial resistance and adverse events, and as such, the impact of PAAF may be maximized if it occurs frequently and focuses not only on antibiotics that are costly or broad-spectrum [8–10].

We evaluated the impact of extending prospective audit and feedback to target all systemic antibiotics on all workdays through sequential, staggered time series analysis on three non-ICU clinical wards of a community teaching hospital.

## Materials and methods

### Study design and setting

The study was conducted at Michael Garron Hospital (MGH), a 490-bed urban community teaching hospital in Toronto, Ontario, Canada. The study took place on three inpatient services: surgery (excluding cardiovascular, vascular, and neurosurgery, as these are not performed at the institution), respiratory medicine, and general internal medicine. Patients were admitted to respective wards based on admission diagnosis. Most patients were admitted from the emergency department or electively post-surgical. Transfer between units was not restricted yet occurred infrequently. Prior to ASP implementation on the study wards, the hospital had an existing ASP utilizing PAAF in the Intensive Care unit, which began in April 2010. However, physicians on the three intervention wards had not received prior exposure to PAAF. The intervention was implemented in a staggered manner across time among the three services. On the surgical wards, the baseline period spanned from July 1, 2009 through June 30, 2010, and the intervention period from July 1, 2010 through September 30, 2014. On the respiratory ward, the baseline period lasted from October 1, 2009 through September 30, 2010, and the intervention period from October 1, 2010 through September 30, 2014. On the medical wards, the baseline period took place from January 1, 2010 through March 31, 2012, and the intervention period from April 1, 2012 through September 30, 2014. In order to compare the patient population before and after intervention implementation, patient characteristics such as age, sex, complexity, and discharge diagnoses, were obtained from the hospital’s decision support system. Patient complexity was measured using the Ontario Ministry’s Health Based Allocation Model Inpatient Grouping (HIG) weights. Patients are assigned HIG weights based on several factors, including age category, number of intervention events, and intervention type [11].

### Intervention

Education was provided to clinicians about the importance and goals of antimicrobial stewardship prior to intervention implementation. This was completed through informal and formal presentations provided by an ASP physician during pre-planned department or division meetings. During these meetings, consultation was sought to determine preferred feedback mechanisms. The ASP team consisted of one of two infectious diseases physicians and two ASP pharmacists. The two ASP pharmacists had prior formal training in antimicrobial stewardship provision. The infectious diseases physicians involved with the ASP started working at the hospital providing infectious disease consultations in 2006 and 2011.

Every weekday at 06:00, automated reports were generated for inpatients on intervention wards who were receiving any systemic antibiotic at that time. These reports were available for the pharmacists in hardcopy in the inpatient pharmacy. The ASP pharmacists were encouraged to be physically present on the ward to review cases with an aim to optimize antimicrobial use according to local published antimicrobial guidelines. The pharmacists were free to discuss changes to antimicrobial management with the clinical team caring for the patient before review with the infectious disease physician. After initial pharmacist evaluation, all pharmacist recommendations for optimization and challenging cases were reviewed with an infectious diseases physician daily. Following this discussion, a note was placed in the patient’s electronic medical record providing specific recommendations regarding optimizing antibiotic therapy. If the suggestions were of a more urgent or challenging manner, an ASP pharmacist or physician would provide the recommendations by phone or in person with the patient’s most responsible physician. The decision to change an antibiotic was left to the discretion of the clinical team caring for the patient.

### Outcome and process measures

Our primary outcome was rates of total systemic antibiotic use (excluding antifungals and antivirals) among patients admitted to surgical, respiratory, and medical wards at MGH in the baseline (before the introduction of PAAF) and intervention (after the introduction of PAAF) periods. In order to account for variable antimicrobial practices in different clinical areas, each ward acted as its own control. For the period between July 1, 2009 and November 30, 2009, paper-based charts were manually reviewed to determine antimicrobial use and calculate days on therapy (DOTs) for systemic antibiotics. On December 1, 2009, the hospital implemented an electronic medical record system, and for the remainder of the study period, the pharmacy’s computerized database was used to determine antibiotic use and calculate DOTs. Total antibiotic use was calculated as the sum of days on therapy for all systemic antibiotics. Antibiotic use was then standardized by patient days (per 1,000).

Secondary outcomes included direct antibiotic costs as well as cases of hospital-acquired *C*. *difficile* infection (HA-CDI). Antibiotic cost data was determined through use of financial charge data (in Canadian dollars) and standardized by patient days. HA-CDI cases were defined using the Ontario Ministry of Health and Long-Term Care case definitions and attributed to wards by the Infection Prevention and Control team based on review of epidemiological links, antibiotic exposure, and time spent in specific locations. HA-CDI rates were standardized per 1,000 patient days.

Process measures included the type and frequency of ASP recommendations over the intervention period. These recommendations were classified according to the Infectious Diseases Society of America guidelines [2], and recommendation acceptance rates were recorded for each clinical area.

### Balancing measures

In order to ensure no unanticipated patient harm as a result of intervention implementation, all-cause mortality, seven-day readmission (pertaining to an emergency department visit or admission to an inpatient unit at MGH) and mean length of stay were measured during the study period. All outcomes were standardized by patient days (per 1,000) on a particular ward per month.

### Statistical analysis

An interrupted time series analysis was performed using segmented linear regression models with auto-correlated error structures that were fit to each of the three ward-specific time series. We assessed changes in the mean level of total systemic antibiotic use pre-post intervention, and in the rate of change of total systemic antibiotic use over time, using a first-order autoregressive model. Each time series was plotted to visually identify patterns of interest. Thereafter, linear regression models with fitted ARIMA errors were used in our respective time series to account for the correlation between repeated observations. The residuals of the fitted models were assessed for autocorrelation using the Durbin-Watson and Ljung-Box tests, and by examining plots of the autocorrelation and partial autocorrelation functions. The model equation is:

$$Y_{t}=\beta_{0}+\beta_{1}*\mathrm{tim}e_{t}+\beta_{2}*\mathrm{interventio}n_{t}+\beta_{3}*time\;after\;intervention_{t}+e_{t}$$

where β_0_ estimates the baseline level of total systemic antibiotic use prior to PAAF, β_1_ estimates the slope prior to PAAF, β_2_ estimates the change in the level of antibiotic use immediately after PAAF, and β_3_ estimates the change in the slope after PAAF. β_1_ and β_3_ are summed to obtain the post-PAAF slope [12]. P-values for the coefficients were calculated using the Wald test statistic.

Changes in total antibiotic costs, rates of HA-CDI, all-cause mortality, length of stay, and seven-day readmission in each study ward before and after PAAF implementation were compared using the Wilcoxon rank-sum test. Patient characteristics in the baseline and intervention periods were compared using the χ^2^ test for categorical variables, and two-tailed Student *t*-test for continuous variables. For all analyses, p-values ≤0.05 were considered significant. Statistical analyses were performed using R version 3.1.1 and were determined *a priori*.

### Ethics

Approval for this study was obtained from the MGH Research Ethics Board. The need for individual patient consent was not required for this quality improvement project.

## Results

### Patient characteristics

Patient characteristics across the study wards during the baseline and intervention periods are presented in Table 1. In the baseline period, a total of 3,969 patients were admitted to the surgical wards (17,090 patient days), 746 were admitted to the respiratory ward (7,160 patient days), and 4,911 were admitted to the medical wards (48,093 patient days). In the intervention period, a total of 16,521 patients were admitted to the surgical wards (64,850 patient days), 3,826 were admitted to the respiratory ward (30,512 patient days), and 5,115 were admitted to the medical wards (49,700 patient days). Most patients on the respiratory and medical wards were admitted from the emergency department. In contrast, the majority of patients on the surgical wards were admitted electively.

**Table 1:** Baseline characteristics of patients in the surgical, respiratory, and medical wards before and after prospective audit and feedback.

	Surgery			Respiratory			Medicine		
Variable	Baseline^a^ (n = 3,969)	Intervention^b^ (n = 16,521)	*p*	Baseline^a^ (n = 746)	Intervention^b^ (n = 3,826)	*p*	Baseline^a^ (n = 4,911)	Intervention^b^ (n = 5,115)	*p*
Age, mean years ± S.D.	57.1 ± 18.2	57.2 ± 17.8	0.761	68.9 ± 16.9	70.5 ± 17.0	0.014	71.0 ± 17.5	72.6 ± 16.8	<0.001
Male sex, %	38.9	40.5	0.063	47.7	46.8	0.639	45	46.5	0.125
Patient days per month, mean ± S.D.	1,424 ± 110	1,272 ± 190	0.001	597 ± 59	636 ± 67	0.061	1781 ± 120	1657 ± 161	0.002
Admitted from									
Emergency	1,543 (39)	5,983 (36)		677 (91)	3,516 (92)		4,733 (96)	4,908 (96)	
Elective	2,316 (58)	9,844 (60)		60 (8)	257 (7)		131 (3)	173 (3)	
Day Surgery	91 (2)	542 (3)		3 (0)	6 (0)		13 (0)	9 (0)	
Clinic	19 (1)	152 (1)	<0.001	6 (1)	47 (1)	0.208	34 (1)	25 (1)	0.074
Diagnosis at hospital discharge, by major clinical category									
Blood & lymphatic system	41 (1)	134 (1)	0.173	8 (1)	35 (1)	0.683	123 (3)	110 (2)	0.24
Circulatory system	20 (1)	70 (0)	0.493	39 (5)	123 (3)	0.007	460 (9)	407 (8)	0.012
Digestive system	710 (18)	2,916 (18)	0.724	14 (2)	86 (2)	0.526	708 (14)	637 (12)	0.004
Ear, nose, mouth & throat	124 (3)	458 (3)	0.231	10 (1)	70 (2)	0.351	53 (1)	61 (1)	0.593
Endocrine system, nutrition & metabolism	274 (7)	1,674 (10)	<0.001	52 (7)	163 (4)	0.001	307 (6)	278 (5)	0.081
Hepatobiliary system & pancreas	117 (3)	450 (3)	0.44	4 (1)	65 (2)	0.017	399 (8)	388 (8)	0.318
Kidney, urinary tract & male reproductive system	409 (10)	1,898 (11)	0.034	6 (1)	46 (1)	0.348	473 (10)	414 (8)	0.007
Musculoskeletal system & connective tissue	709 (18)	2,697 (16)	0.019	2 (0)	28 (1)	0.143	225 (5)	310 (6)	<0.001
Nervous system	8 (0)	31 (0)	0.857	8 (1)	44 (1)	0.855	537 (11)	665 (13)	0.001
Respiratory system	261 (7)	1,276 (8)	0.014	524 (70)	2,726 (71)	0.579	351 (7)	197 (4)	<0.001
Skin, subcutaneous tissue & breast	75 (2)	275 (2)	0.326	5 (1)	24 (1)	0.856	171 (3)	178 (3)	0.996
Multi-systemic or unspecified site infections	9 (0)	36 (0)	0.915	24 (3)	96 (3)	0.27	119 (2)	201 (4)	<0.001
Other^c^	1,212 (31)	4,606 (28)	<0.001	50 (7)	320 (8)	0.128	985 (20)	1,269 (25)	<0.001
HIG weight, mean ± S.D.	1.4 ± 1.8	1.4 ± 2.4	0.291	1.8 ± 6.5	1.9 ± 5.2	0.611	1.7 ± 3.3	1.7 ± 2.6	0.625

Unless otherwise specified, data are no. (%) of patients

Abbreviations: S.D., standard deviation; HIG, Health Based Allocation Model Inpatient Group

^a^ July 1, 2009 –June 30, 2010 in the surgical wards; October 1, 2009 –September 30, 2010 in the respiratory ward; January 1, 2010 –March 31, 2012 in the medical wards

^b^ July 1, 2010 –September 30, 2014 in the surgical wards; October 1, 2010 –September 30, 2014 in the respiratory ward; April 1, 2012 –September 30, 2014 in the medical wards

^c^ Includes burns; diseases of the eye; female reproductive system; mental diseases & disorders; miscellaneous & ungroupable data; other reasons for hospitalization; pregnancy & childbirth; significant trauma, injury, poisoning & toxic effects of drugs

In each study ward, gender and patient complexity were similar in the baseline and intervention periods. On the respiratory and medical wards, the mean age of patients significantly increased in the intervention period. The majority of discharge diagnoses remained similar after ASP implementation. On the medical wards, however, the proportion of patients with diagnoses categorized as “other” significantly increased, predominately due to an increase in patients diagnosed with mental illnesses (6% and 9% in the pre and post PAAF periods, respectively). Also, the proportion of patients diagnosed with multi-systemic or unspecified site infections increased significantly from 2% to 4% in the intervention period on the medical wards.

### Antibiotic recommendations

On the surgical wards, a total of 1,176 antibiotic recommendations were made over the 51 month intervention period (mean 23.0 recommendations/month). Data on acceptance or rejection were available for 1,112 (94.5%) recommendations, of which 93.0% were accepted. After prospective audit and feedback implementation on the respiratory ward, 1,529 antibiotic recommendations were made (mean 31.8 recommendations/month). Data on acceptance or rejection were available for 1,495 (97.8%) recommendations, of which 97.5% were accepted. On the medical wards, 1,794 recommendations were made over the 30 month intervention period (mean 59.8 recommendations/month). Data on acceptance or rejection were available for 1,720 (95.9%) recommendations; of these, 97.3% were accepted. The most common recommendations made over the study period included optimizing the duration of antibiotic therapy (33.1%), recommendations to discontinue antibiotic therapy (22.4%), and changes in the route of administration (16.2%). The types and frequency of antibiotic recommendations by ward are presented in Table 2.

**Table 2:** Type and frequency of antibiotic recommendations made after the implementation of prospective audit and feedback on the surgical, respiratory, and medical wards.

Type of Recommendation	Surgery no. (%)	Respiratory no. (%)	Medicine no. (%)
Intravenous to per os step-down	129 (11.0)	373 (24.4)	229 (12.8)
Dose adjustment for renal or hepatic impairment	25 (2.1)	32 (2.1)	36 (2.0)
Dose optimization for indication	114 (9.7)	76 (5.0)	111 (6.2)
Duration optimization	305 (25.9)	606 (39.6)	576 (32.1)
Recommendation to discontinue therapy	324 (27.6)	269 (17.6)	413 (23.0)
Recommendation to de-escalate therapy	103 (8.8)	66 (4.3)	164 (9.1)
Recommendation to change to a broader agent or different agent for empiric coverage	125 (10.6)	69 (4.5)	140 (7.8)
Suggest infectious diseases consultation	36 (3.1)	28 (1.8)	64 (3.6)
Suggest intervention/imaging	15 (1.3)	10 (0.7)	61 (3.4)
Total Recommendations	1,176	1,529	1,794

### The impact of stewardship on total systemic antibiotic use

**Fig 1:**
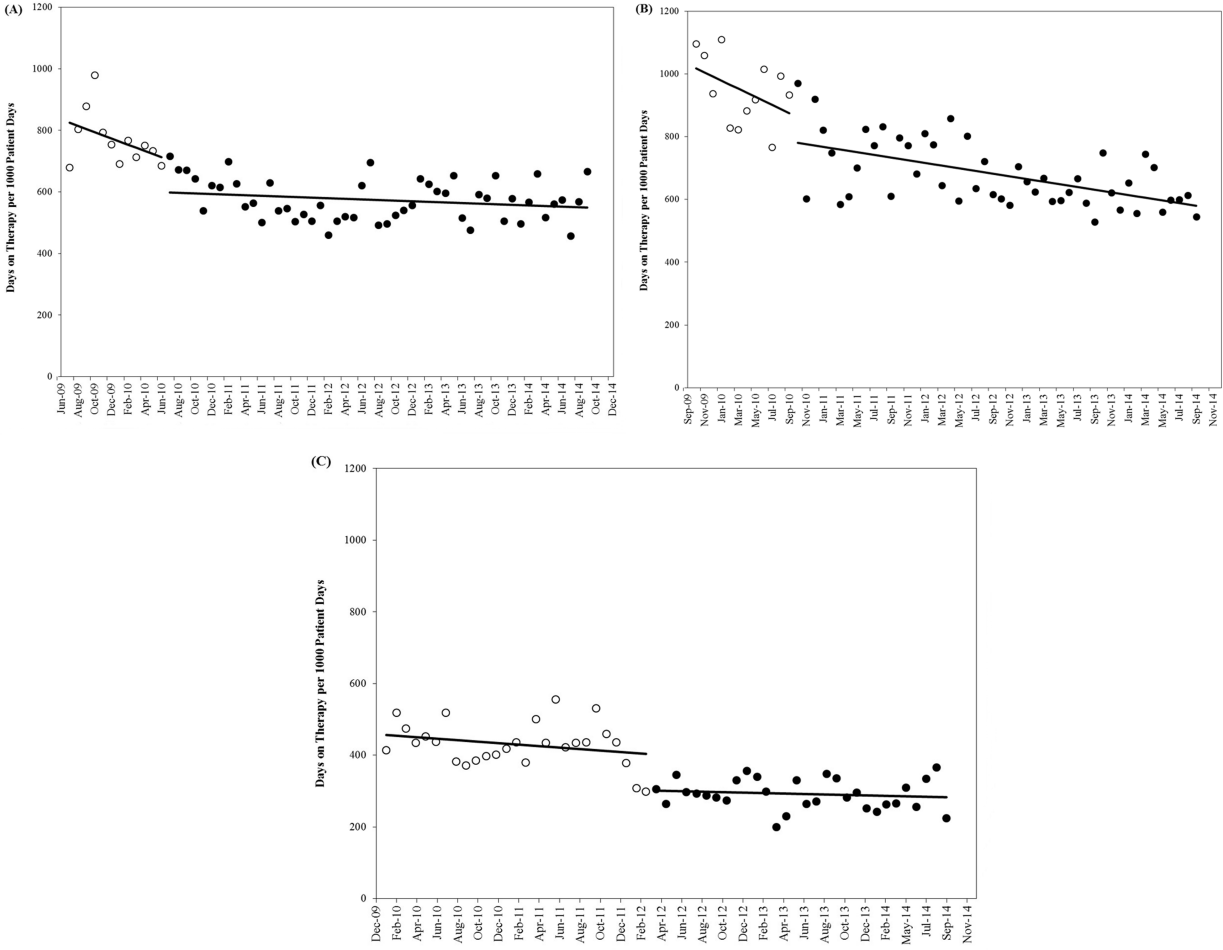
Antibiotic use in the study wards before and after prospective audit and feedback implementation. A) Surgical wards. B) Respiratory ward. C) Medical wards. The unfilled circles represent the baseline period, and the filled circles represent the intervention period. For each study ward, linear trend lines were fit to the data for the baseline and intervention periods.

On the surgical wards, total antibiotic use decreased from 765 DOTs per 1,000 patient days in the baseline period to 572 DOTs per 1,000 patient days after PAAF initiation. The results of the interrupted time series model (Table 3) demonstrated that although total systemic antibiotic use showed a non-significant declining trend before the introduction of PAAF (p = 0.125), antibiotic use was significantly decreased by 100 DOTs per 1,000 patient days (p = 0.049) immediately after intervention implementation, signifying a 12% reduction. The overall trend in total systemic antibiotic use was stable in the months following the introduction of PAAF (p = 0.173) (Fig 1A).

**Table 3:** Results of segmented regression analyses evaluating the impact of prospective audit and feedback on systemic antibiotic use^a^ in the surgical, respiratory, and medical wards.

	Surgery			Respiratory			Medicine		
	Coefficient	S.E.	*p*	Coefficient	S.E.	*p*	Coefficient	S.E.	*p*
Baseline antibiotic use	826.37	50.79		1022.50	45.58		459.79	24.34	
Monthly slope of antibiotic use before ASP	-10.34	6.74	0.125	-11.64	6.23	0.061	-2.25	1.52	0.139
Change in antibiotic use at time of ASP initiation	-99.76	50.59	0.049	-100.06	45.70	0.029	-90.69	33.03	0.006
Change in slope of antibiotic use after ASP	9.31	6.82	0.173	7.45	6.26	0.234	1.25	2.02	0.537

Abbreviations: S.E., standard error

^a^ Antibiotic use measured in days on therapy per 1,000 patient days

On the respiratory ward, total antibiotic use decreased from 946 DOTs per 1,000 patient days in the baseline period to 678 DOTs per 1,000 patient days after the introduction of PAAF. Time series modelling showed that there was a non-significant declining trend in total systemic antibiotic use prior to the introduction of the intervention (p = 0.061). Immediately after the implementation of PAAF, total systemic antibiotic use was significantly decreased by 100 DOTs per 1,000 patient days (p = 0.029), a 10% reduction. In the intervention period, the trend in total systemic antibiotic use on the respiratory ward demonstrated a non-significant decline (p = 0.234) (Fig 1B).

On the medical wards, total antibiotic use decreased from 430 DOTs per 1,000 patient days in the baseline period to 293 DOTs per 1,000 patient days after the introduction of PAAF. Time series modelling showed that there was a non-significant decline in total systemic antibiotic use during the baseline period (p = 0.139). Immediately following the introduction of the stewardship program, total antibiotic use was significantly decreased by 91 DOTs per 1,000 patient days (p = 0.006), representing a 20% reduction. In the months following the introduction of PAAF, the trend in total systemic antibiotic use on the medical wards stabilized (p = 0.537) (Fig 1C).

### Changes in the use of individual antibiotics

**Fig 2:**
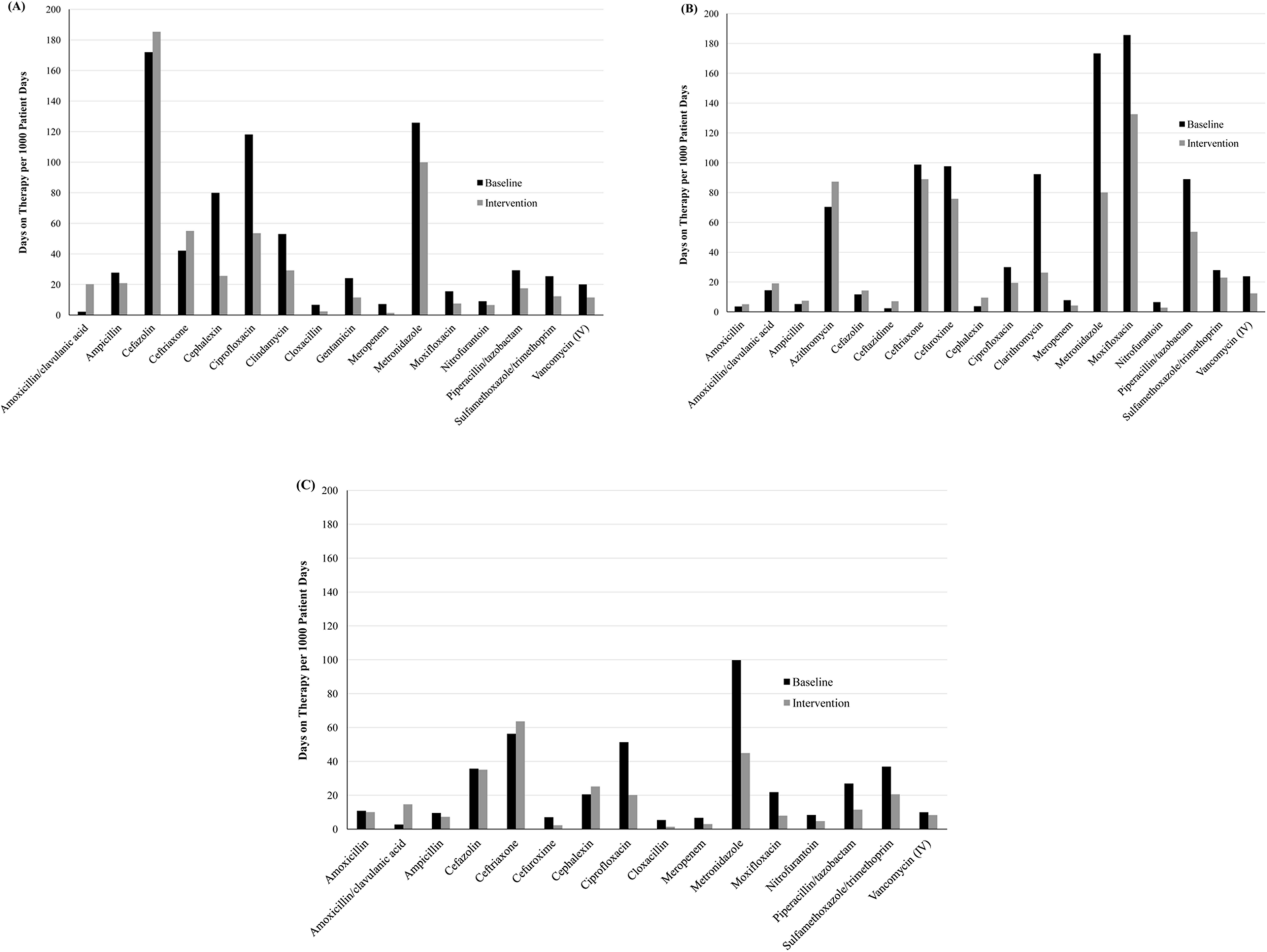
Utilization of individual antibiotics in the study wards before and after prospective audit and feedback implementation. A) Surgical wards. B) Respiratory ward. C) Medical wards. Only antibiotics for which the mean baseline or intervention use was ≥5 DOTs per 1,000 patient days are displayed.

Fig 2 displays antibiotics for which the mean utilization during the baseline or intervention period was ≥5 DOTs per 1,000 patient days. Of the 16 antibiotics that met this criteria in the surgical wards, all except amoxicillin/clavulanic acid, cefazolin, and ceftriaxone showed decreases in utilization during the intervention period. Of 18 antibiotics that met the criteria in the respiratory ward, small increases in utilization were observed for amoxicillin, amoxicillin/clavulanic acid, ampicillin, azithromycin, cefazolin, ceftazidime, and cephalexin. Utilization of the other 11 antibiotics decreased after PAAF initiation. Of 16 antibiotics in the medical wards, utilization increased for amoxicillin/clavulanic acid, ceftriaxone, and cephalexin, while the use of all other antibiotics showed a decrease.

### Antibiotic costs

On the surgical wards, daily antibiotic costs were reduced by 35% after the introduction of antimicrobial stewardship, from $5.64 to $3.66 per patient day (p<0.001). Costs decreased from $9.40 to $5.51 per patient day following PAAF on the respiratory ward, representing a 41% reduction (p<0.001). After PAAF was implemented on the medical wards, antibiotic costs declined from $3.35 to $2.17 per patient day, indicating a 35% decrease (p<0.001).

### Hospital-acquired *C*. *difficile* infection

The incidence of HA-CDI demonstrated a non-significant declining trend after the implementation of PAAF on the surgical wards, from 0.82 to 0.39 cases per 1,000 patient days (p = 0.174), on the respiratory ward from 2.37 to 0.82 cases per 1,000 patient days (p = 0.060), and on the medical wards from 0.79 to 0.44 cases per 1,000 patient days (p = 0.079).

### Patient outcomes

Changes in clinical outcomes across the study periods are presented in Table 4. On the surgical and respiratory wards, mortality, readmission, and length of stay did not change significantly following the introduction of PAAF. On the medical wards, seven-day readmission increased significantly in the intervention period, from 4.62 to 5.63 per 1,000 patient days (p = 0.043). In contrast, mortality was significantly reduced, from 7.40 to 5.01 per 1,000 patient days (p = 0.001).

**Table 4:** Patient outcomes before and after prospective audit and feedback in the surgical, respiratory, and medical wards.

	Surgery			Respiratory			Medicine		
Patient Outcomes	Baseline	Intervention	*p*	Baseline	Intervention	*p*	Baseline	Intervention	*p*
Mortality	0.99	0.97	0.763	11.45	12.22	0.437	7.40	5.01	0.001
Readmission	5.38	6.93	0.073	6.70	5.60	0.617	4.62	5.63	0.043
Mean length of stay	4.73	4.25	0.052	9.60	8.46	0.156	10.23	10.30	0.512

Unless otherwise specified, data are no. per 1,000 patient days

## Discussion

We evaluated the impact of prospective audit and feedback targeting all systemic antibiotics in three non-ICU services of a large community hospital. The intervention was associated with statistically significant, immediate reductions in total antibiotic use across the three locations, despite differences in patient populations and the timing of intervention initiation. After PAAF was introduced on the surgical, respiratory, and medical wards, total systemic antibiotic use was reduced by 12%, 10%, and 20%, respectively and these reductions were sustained for up to 50 months following the implementation of the intervention. PAAF was also associated with substantial reductions in antibiotic costs and a non-significant declining trend in the incidence of nosocomial *C*. *difficile* infection. There were no significant changes to patient outcomes on the surgical and respiratory wards following intervention initiation. On the medical wards, however, readmission increased, while mortality decreased.

Although other studies have demonstrated reductions in total antibiotic utilization after the implementation of antimicrobial stewardship, our study adds substantially to the existing literature. We staggered intervention implementation across the study wards, allowing us to evaluate the independent impact of our intervention on wards with different patient characteristics. There is limited literature evaluating the differential impact of PAAF in different clinical settings outside of the ICU, yet in our study we demonstrated that our intervention is generalizable to general surgical wards, as well as general medical and respiratory wards. Current literature evaluating the impact of comprehensive ASPs on surgical wards is limited and have shown variable success [13–15]. Our intervention led to a 12% reduction in total systemic antibiotic use in the general surgery wards, similarly to the study by Sartelli et al. [15], which demonstrated an 18.8% decrease in a combined general and emergency surgery unit. The success of our intervention on the surgical wards was likely facilitated by the similar philosophies of non-restriction as the Sartelli et al. [15] study. By conducting PAAF on all inpatient antibiotic orders, we maximized the potential for educational opportunities and engagement of surgical staff. The magnitude of our reduction on other units is also similar to prior studies evaluating the impact of antimicrobial stewardship programs. In the studies by Borde et al. [5] and Boyles et al. [6], there was a decrease in total antibiotic use of 14.2% and 19.6%, respectively, in the medical wards where the interventions were implemented. Comparing the reduction in total antibiotic use between studies is challenging, as the magnitude of impact is likely heavily dependent on baseline use and the method of measuring antimicrobial consumption [3]. However, the ability to target all antibiotics on all days, as we did in our intervention, acted to limit the often seen increase in non-targeted antibiotics leading to the so called “squeeze the balloon” phenomenon. In our study, we observed increases in the utilization of cephalosporins and beta-lactam/beta-lactamase inhibitor antibiotics as was seen in prior studies, but at a lower magnitude. Through PAAF, we preferentially used cephalosporins and beta-lactam/beta-lactamase inhibitor antibiotics over fluoroquinolone-based regimes.

Determining which antibiotics to target and how frequently to provide PAAF is often an arbitrary decision at the initiation of an ASP with little evidence to guide these program decisions. Many published ASP interventions used weekly rounds but provided little direction to clinicians outside of guidelines or education between rounds [3]. As such, several studies have demonstrated reductions among targeted antibiotics, but have shown little impact among non-targeted or total antibiotic use. For instance, Palmay et al’s [8] antimicrobial stewardship program targeted patients on any of 9 antibiotics on days 3 and 10 of therapy. There were no reductions in non-targeted or total antibiotic use among patients qualifying for stewardship, nor were there reductions in targeted, non-targeted, or total antibiotic use among all admitted patients. Similarly, Yeo et al. [9] performed PAAF on broad-spectrum antibiotics among patients in a hematology-oncology unit after 3 days of therapy, and found no decrease in overall antibiotic consumption. In a study by Cheng et al. [16], stewardship was conducted daily, but only intravenous broad-spectrum antibiotics were targeted and as a result, no reductions in total antibiotic use were observed. Our study provides evidence of the success of antimicrobial stewardship when all systemic antibiotics are targeted among inpatients receiving any duration of antibiotic therapy. If PAAF can reduce the use of targeted antibiotics, then stewardship programs should extend the focus to target all systemic antibiotics.

A major factor predicting the success of an ASP is physician engagement. The success of PAAF demonstrated in the literature is likely related to the combined impact of the intervention to optimize antimicrobial use as well as the additional benefit of providing the opportunity for case-specific, real-time education with antibiotic prescribers. Each episode of PAAF is a learning opportunity to improve prescribing behaviors moving forward [17]. By conducting PAAF daily on all systemic antibiotics, as we did in our study, we were able to maximize the potential for interaction with providers and the ASP team. During our intervention period, there were between 23.0 and 59.8 opportunities per month on wards for real-time, case-based education. Examination of the distribution of ASP recommendation types across the three intervention wards showed that there was relative consistency, with duration optimization, recommendations to discontinue treatment, and intravenous to oral step-down being the most common. None of these recommendation types would be specific to broad-spectrum or expensive antimicrobials. Based on the distribution of antibiotic use at our hospital, the majority of these recommendations were likely directed at antibiotics that would not meet PAAF review criteria of many ASPs.

In our study, we observed a non-significant declining trend in the incidence of hospital-acquired *C*. *difficile* infection following the introduction of stewardship. The literature has shown mixed results concerning the impact of stewardship on nosocomial *C*. *difficile* infection; some studies have shown a beneficial effect [18–21], while another study showed a null effect [8]. A recent systematic review and meta-analysis found no significant change in the incidence of hospital-acquired *C*. *difficile* after ASP implementation. This finding was, however, only based on three studies, and publication bias in this area was found to be significant [3]. In our study, the lack of statistical significance may be due to the small absolute number of *C*. *difficile* infections, such that there may be insufficient power to detect changes. Although reductions in hospital-acquired *C*. *difficile* infection rates did not reach statistical significance, the consistent decrease in *C*. *difficile* infection on all study wards after intervention implementation suggests that further study in this area may be warranted.

On the surgical and respiratory wards, patient outcomes did not change significantly following the introduction of antimicrobial stewardship. On the medical wards, however, readmission significantly increased in the intervention period, while mortality demonstrated a significant decrease. It is worthy to note, however, that we did not attribute mortality, readmission, or length of stay specifically to diagnoses related to infectious diseases, and thus, changes in these metrics could be due to factors other than stewardship. The increased readmission rate on the medical wards could be related to the increase in the proportion of patients with a mental health diagnosis during the intervention period, as the readmission rate for patients with a mental health diagnosis has been noted to be greater [22]. While the significant decrease in mortality seen on the medical wards may demonstrate a potential benefit of the daily PAAF used in our study, we cannot disregard that there were other unrelated interventions on the medical wards during the same time period, which could have also contributed to the changes in patient outcomes on this ward. Future research should aim to assess the impact of PAAF on clinical outcomes attributed specifically to those patients with infectious diseases.

There are several limitations to our study. As our study was not a randomized trial, there were some changes in the characteristics of the study populations between the baseline and intervention periods that could have potentially contributed to changes in antibiotic use patterns. Patient complexity, however, remained similar, and the magnitude of the changes in discharge diagnoses were small. Furthermore, this study was not a prospectively designed randomized controlled trial, and therefore issues related to selection bias or co-interventions could impact the validity of the findings. The potential for bias was limited, however, by the implementation of our intervention at different times in each inpatient service. The lack of potential confounding is demonstrated by the similarity in direction and magnitude of changes in antibiotic use, costs, and nosocomial *C*. *difficile* infection across wards. In our study, the medical wards had a longer baseline relative to the surgical and respiratory wards, which may have improved our ability to detect significant changes in antibiotic use on this ward. Electronic antibiotic use data became available in January 2010, and thus we used all available data to conduct analyses in the medical wards. In contrast, it was not realistic to use this baseline for the surgical and respiratory wards, as antimicrobial utilization data would need to be manually extracted from paper-based charts. Despite the longer baseline period, the results in the medical wards were similar to those found in the surgical and respiratory wards. Sensitivity analyses restricting the baseline period to the same length as the surgical and respiratory wards did not impact the significance of our findings. In addition, by restricting our analysis to total antibiotic use in specific wards, rather than total antibiotic use throughout the hospital, we may have disregarded antibiotic use that patients had on non-study wards. Lastly, we did not perform a detailed cost analysis to evaluate whether targeting all systemic antibiotics provided cost advantages over targeting only expensive or broad-spectrum antibiotics. Although cost avoidance is an important factor to assist in the financial justification for dedicated resources to provide stewardship, the goal of antimicrobial stewardship is to optimize antimicrobial use and resultant patient outcomes, not to save money.

## Conclusions

Our study demonstrated the sustainable impact of an antimicrobial stewardship program using prospective audit and feedback, targeting all systemic antibiotics among patients on any duration of antibiotic therapy in three non-ICU settings of a large community hospital. There were substantial reductions in antibiotic utilization and costs, and a non-significant declining trend in the incidence of hospital-acquired *C*. *difficile*. However, the impact of antimicrobial stewardship programs on clinical outcomes needs further study. These results illustrate that antibiotic stewardship can be successfully expanded outside of the critical care setting, and that the focus should shift from targeting only expensive and/or broad-spectrum agents to optimizing the use of all systemic antibiotics on all days. Each episode of PAAF acts as an opportunity to educate clinicians about the importance of antimicrobial optimization and assists with prescriber engagement. At a time when concerns about antibiotic misuse and resistance are rising, it is important that funding agencies allocate sufficient resources to stewardship programs to facilitate expanding the role of PAAF.

## Supporting information

S1 Dataset Data analyzed for the study. (XLSX)
